# 

**Published:** 1914-04-18

**Authors:** 


### CONTENTS.

PAGE

**Married Women Doctors and their Appointments** ... ... ... ... ... ... ... ... 57

**The Modern Medical Bureaucrat** ... ... ... 58

**Hospital and Institutional News** ... ... ... 59

The Doctors' Wives Association—Industrial Assurance—A Crucial Moment at Cardiff—Maternity Beds to be Opened—The Human Side of Hospital Finance—The Hospital Secretary's House—A Lesson from Ulster at Worthing—Salaries and the Saturday Fund—The Treatment of Uninsured Tuberculous Patients—Altering the Rules at Gravesend Hospital—Summer Severities at Southwark Infirmary—Infirmaries and the Insurance Act—A Medical Revolution in Madeira—Voluntary *versus* Municipal Dispensaries — Doctor as Prisons Commissioner—A Hove Public Medical Service—Doctor's Precaution against Premature Burial—Charles Dickens on Ward Work—The Chaplain's Department—The Cicely Northcote Trust—Doctor, Pulpit, and Patient—A Radium Institute for Glasgow—The Blackpool Hospital Deadlock—The Duty of the Subscribers—Hospitals and Social Functions—Lord St. Aldwyn on the Voluntary System—This Week's Drug Market.

PAGE

**The Treatment of Puerperal Sepsis in General Practice** ... ... ... ... ... ... ... 65

**Nervous Diseases in School Children** ... ... 67

A Rare Problem of School Work.

[*See over.*]

### CONTENTS—(*continued*).

| | PAGE |
|---|---|
| **The Problem of the Advanced Consumptive** ... | 68 |
| An Alternative to Institutional Segregation. | |
| **Reports on Hospitals of the United Kingdom**— By SIR HENRY BURDETT, K.C.B., K.C.V.O | 69 |
| General Kent and Canterbury Hospital. | |
| **Buildings Contemplated** ... ... ... ... | 70 |
| **Hospital Architecture and Construction** ... | 71 |
| Sanatoria Buildings at £88 per Bed. | |
| **Round the World** ... ... ... ... ... | 73 |
| Fellow-Workers and their Doings. | |
| **Some Chapels of Famous Hospitals** ... ... | 74 |
| The Central Point of the Chaplain's Work. | |
| **The Editor's Letter-Box** ... ... ... ... | 77 |
| Women Doctors and the London County Council —Non-Panel Men and the Medical Directory —The Artificial Pneumothorax Association and Journal—Radium in the Treatment of Deafness—Insuring Babies' Lives. | |
| **Appointments** ... ... ... ... ... ... | 78 |
| **Doctors' Wills** ... ... ... ... ... ... | 78 |
| **Personalia** ... ... ... ... ... ... ... | 78 |
| **A Mental Hospital Sketch** ... ... ... ... | 78 |
| **Women Doctors and the London County Council** ... ... ... ... ... | 79 |
| The Debate on the Marriage Question. | |
| **The Patients' Column** ... ... ... ... ... | 80 |
| **Gifts and Bequests** ... ... ... ... ... | 80 |
| **The Book Market** ... ... ... ... ... | 80 |
| **The Doctors' Wives Association** ... ... ... | 80 |
| **Practical Points in Hospital Mechanics** ... | 81 |
| Lightning Conductors and their Tests. | |
| **The National Medical Union (Official)** ... ... | 82 |
| Report of Council Meeting. | |
| The Medical Benefit "Surplus Funds." | |
| **The Institutional Worker** ... ... (Suppl.) | 1 |
| Annual Painting and Cleaning. | |
| Questions for April. | |
| Questions Invited. | |
| The Sick and in Need. | |
| Employment and Training. | |

